# Identification of Functional SNPs in CTGF Regulating Fat Deposition in Broilers

**DOI:** 10.3390/cells15211930

**Published:** 2026-10-23

**Authors:** Jiamei Zhou, Yan Song, Cheng Chang, Hengcong Chen, Zhiping Cao, Yumao Li, Peng Luan, Wenpeng Zhang, Hui Li, Hui Zhang

**Affiliations:** 1 College of Animal Science and Technology, Northeast Agricultural University, Harbin 150030, China; 2 Key Laboratory of Chicken Genetics and Breeding, Ministry of Agriculture and Rural Affairs, Harbin 150030, China; 3 Key Laboratory of Animal Genetics, Breeding and Reproduction, Education Department of Heilongjiang Province, Harbin 150030, China

**Keywords:** broiler, fat deposition, CTGF, SNP, functional analysis

## Abstract

Broilers are one of the most important sources of animal protein worldwide due to their rapid growth rate and high feed efficiency. However, excessive abdominal fat deposition in broilers reduces feed efficiency and carcass quality, resulting in substantial economic losses to the poultry industry. In previous studies, we have demonstrated that connective tissue growth factor (CTGF) is a key gene affecting chicken fat deposition, but its specific molecular mechanism is still unclear. This study aimed to identify functional single-nucleotide polymorphisms (SNPs) within the CTGF gene associated with abdominal fat deposition and to elucidate their potential regulatory mechanisms in broilers. Whole-genome resequencing data from the 19th generation of broiler lines divergently selected for abdominal fat content were used to identify SNPs within CTGF and its flanking regions (±3 kb). Association analyses between SNPs and abdominal fat traits, including abdominal fat weight (AFW) and abdominal fat percentage (AFP), were subsequently performed. Seven SNPs were significantly associated with AFW and AFP, among which four were further validated as functional SNPs by Reverse Transcription Quantitative Polymerase Chain Reaction (RT-qPCR) and luciferase reporter assay, including g + 516T > C, g + 954T > C, g − 982A > G, and g *2702A > T. The results of RT-qPCR indicated that the exonic SNPs g + 516T > C and g + 954T > C may regulate CTGF expression by altering mRNA secondary structure stability. The SNP g − 982A > G, located in the 5′ flanking region, may affect CTGF transcriptional activity through allele-dependent responses to transcription factors CCAAT/enhancer-binding protein alpha (C/EBPα), upstream stimulatory factor 1 (USF1), and MYC-associated factor X (MAX). In addition, the SNP g *2702A > T, located in the 3′ flanking region, may influence CTGF regulation by affecting a potential gga-miR-1812-5p-responsive element. These findings improve our understanding of the genetic mechanisms underlying abdominal fat deposition and provide potential molecular markers for marker-assisted selection for abdominal fat content in broiler breeding programs.

## 1. Introduction

Chicken meat is one of the most important sources of animal protein for humans, and fast-growing, white-feathered broilers are the major contributors to global chicken production. Over the past decades, intensive genetic selection for increased meat yield and rapid growth rate has achieved remarkable success in improving broiler production performance. Nevertheless, long-term genetic selection for improved growth performance has also brought unintended consequences, including a higher susceptibility to physiological disorders and metabolic diseases in broilers, such as excessive fat deposition, ascites syndrome, and sudden death syndrome [1]. Excessive fat deposition, in particular, represents a major challenge in current broiler breeding programs. This problem negatively affects feed conversion efficiency and reproductive performance, compromises carcass quality, increases processing waste, and leads to considerable economic losses in the poultry industry [2,3,4]. Therefore, breeding high-quality broilers with reduced abdominal fat deposition has become an important objective in poultry breeding programs. Abdominal fat traits in chickens are complex quantitative traits influenced by multiple genetic variations, and their genetic regulatory mechanisms remain highly complex. Single-nucleotide polymorphisms (SNPs) represent an important kind of genetic variation and have been widely used to identify loci associated with economically important traits in chickens. Previous studies have identified numerous SNPs associated with fat-related traits in chickens [5,6,7,8]. However, most of these loci were identified through genome-wide association analyses using large-scale genomic data and remain statistical associations rather than validated causal variants. Their biological functions and regulatory mechanisms underlying fat deposition are still largely unclear, which limits their practical application in molecular breeding.

Increasing evidence suggests that functional SNPs can directly influence phenotypic variation by altering transcription factor binding activity, chromatin accessibility, mRNA stability, and RNA secondary structure, thereby regulating gene expression. Therefore, identifying functional and potentially causal SNPs is essential for elucidating the genetic basis of fat deposition and developing reliable molecular markers for marker-assisted selection and genomic selection in broilers.

Connective tissue growth factor (CTGF) was first identified in the conditioned medium of human umbilical vein endothelial cells in 1991 and is a cysteine-rich secreted peptide [9]. CTGF is involved in the regulation of multiple cellular functions, including extracellular matrix formation, cell adhesion, migration, mitosis, and apoptosis [10]. These properties enable CTGF to exert diverse biological effects during cell growth, differentiation, pathological conditions such as tumorigenesis and fibrosis, as well as wound healing processes [11]. Several studies have demonstrated that SNPs within the CTGF gene are significantly associated with traits and diseases such as diabetes, nephropathy, and colon cancer [12,13].

In our previous study, we found that CTGF could inhibit chicken preadipocyte differentiation [14]. However, the sequence variation within the CTGF locus and its association with chicken abdominal fat deposition have not been systematically characterized. In particular, it remains unclear whether SNPs located within CTGF and its flanking regions affect adipogenesis by modulating CTGF expression. In the present study, we used resequencing data from fat and lean male broiler lines to identify SNPs within the CTGF gene and its 3 kb flanking regions, and performed association analyses between SNPs and abdominal fat traits. Furthermore, the potential functions and regulatory mechanisms of candidate SNPs were investigated using association analysis, expression analysis, transcription factor binding prediction, dual-luciferase reporter assays, and preadipocyte differentiation assays. Our findings provide new insights into the roles and molecular mechanisms of functional CTGF SNPs in adipogenesis and may facilitate the improvement of chicken abdominal fat traits through marker-assisted selection.

## 2. Materials and Methods

### 2.1. Experimental Populations

Northeast Agricultural University Broiler Lines (NEAUHLF) have been established since 1996, using abdominal fat percentage (AFP) = abdominal fat weight (AFW)/body weight at 7 weeks of age (BW7) and plasma very-low-density lipoprotein (VLDL) levels as selection criteria. All birds are kept under similar environmental conditions and have free access to feed and water. Commercial corn–soybean-based diets that met all NRC (1994) requirements were provided in the study.

In the study, BW7 was recorded before slaughter. After slaughter and evisceration, abdominal fat was carefully dissected and weighed to obtain AFW. AFP was calculated as AFW divided by BW7 × 100.

### 2.2. Genotyping of SNPs

In our previous study, whole-genome resequencing was performed on 330 seven-week-old male broilers from the 19th generation of the NEAUHLF, including 160 individuals from the lean line and 170 individuals from the fat line [15]. Genome-wide SNP marker information was obtained through resequencing analysis. In the present study, subsequent functional analyses were conducted on SNPs located within the CTGF gene and its upstream and downstream 3 kb flanking regions.

### 2.3. Plasmid Construction

Based on the FASTA sequences of chicken CTGF (GenBank: NC _ 052534.1), upstream stimulatory factor 1 (USF1) (GenBank: NC _ 052556.1), and MYC-associated factor X (MAX) (GenBank: NC _ 052536.1) genes obtained from the NCBI, primers were designed using Primer Premier 5.0 software. Site-directed mutagenesis primers were designed using the CE Primer Design platform provided by Vazyme (https://crm.vazyme.com/cetool/singlepoint.html accessed on 1 March 2025). The primer sequences are listed in Table S1.

Different primer pairs designed above were used to amplify DNA fragments containing the SNPs. Eukaryotic expression plasmids g + 516T > C mutant-type (MT) and g + 954T > C MT were constructed using site-directed mutagenesis methods. Wild-type (WT) and MT reporter gene plasmids, including pGL3 g − 982A > G, pGL3 g *1380A > G, pGL3 g *2702A > T, pGL3 g *3319G > A, and pGL3 g *3337T > G, were generated by homologous recombination and site-directed mutagenesis methods. The CCAAT/enhancer-binding protein alpha (C/EBPα) eukaryotic expression vector was synthesized by Azenta Biotechnology Co., Ltd. (Suzhou, China).

### 2.4. Cell Culture and Induced Differentiation

Chicken preadipocyte ICP2 was constructed in our laboratory [16]. All cells were cultured in basic medium (DMEM/F12, Gibco, Grand Island, NY, USA) containing 10% fetal bovine serum (FBS, Absin, Shanghai, China) and 1% penicillin–streptomycin (Gibco, Grand Island, NY, USA). The cells were inoculated into a 25 cm^2^ culture flask and cultured in a 37 °C, 5% CO_2_ incubator.

Cells were inoculated onto the cell plate. When the confluence reached 60%, the medium was removed, and the oleic acid (Sigma, St. Louis, MO, USA) induction medium with a concentration of 200 μM was added to induce cell differentiation. The oleic acid medium was replaced every 24 h.

### 2.5. Luciferase Assays

For transfection of the luciferase reporter plasmid, cells were seeded in 24-well plates at 70–80% confluence and transfected with pGL3-Basic Vector containing the pGL3 g − 982A > G, pGL3 g *1380A > G, pGL3 g *2702A > T, pGL3 g *3319G > A, pGL3 g *3337T > G, and pRL-TK Renilla luciferase vector (Promega, Wi. Madison, WI, USA) as an internal control using Lipofectamine 3000 (Invitrogen, Carlsbad, CA, USA). For the co-transfection of the luciferase reporter plasmid and the expression plasmid, cells were seeded in 24-well plates, transfected with pGL3-Basic Vector containing the pGL3 g − 982A > G, the pRL-TK Renilla luciferase vector, and pCMV-USF1, pCMV-MAX or pCMV-C/EBPα expression vector using Lipofectamine 3000 (Gibco, Grand Island, NY, USA). For the g.*2702A > T locus, DNA fragments carrying the different alleles were cloned into the psiCHECK-2 vector between the XhoI and NotI restriction sites. The recombinant reporter constructs were generated by Suzhou Anshengda Biotechnology Co., Ltd. (Suzhou, China). and subsequently used for dual-luciferase reporter assays. Cells were collected and luciferase activity was measured using the dual-luciferase reporter assay system (Promega). Firefly luciferase activity was normalized to Renilla luciferase.

### 2.6. RNA Extraction, Reverse Transcription and RT-qPCR

Total RNA was extracted from tissues (100 mg each) and cells using an RNAiso reagent kit (TaRaKa, Beijing, China). First-strand cDNA synthesis was performed with 1 μg of total RNA (TaKaRa, Beijing, China). SYBR Green Premix Pro Taq HS qPCR Kit (Accurate Biotechnology, Changsha, China) was used for Reverse Transcription Quantitative Polymerase Chain Reaction (RT-qPCR). Reaction conditions were as follows: 95 °C for 30 s followed by 40 cycles at 95 °C for 5 s and 60 °C for 30 s. Data analysis was performed using the average threshold cycle (ct) of each sample. The relative expression level of the gene was calculated using the 2^−ΔΔct^ method. The internal reference gene was TATA box-binding protein (TBP). The primers used for RT-qPCR are shown in Table S1.

### 2.7. Oil Red O Staining and Extraction

The medium in the cell plate was removed and washed with PBS 3 times. Each well was fixed for 30 min at 4 °C after adding 4% paraformaldehyde. After discarding the fixative, the cell wells were washed with PBS for 3 times. Subsequently, they were stained with Oil red O (Sigma, St. Louis, USA) working solution (Oil red O stock solution:distilled water = 3:2) at room temperature for 15 min. After removing the staining solution and washing with PBS for 3 times, 60% isopropanol was added to each well for 10–20 s. Washed with PBS 3 times after removing the isopropanol. Finally, the staining was observed using an inverted microscope. After photographing, the Oil red O was dissolved in 100% isopropanol, and the OD value was measured at 510 nm with an enzyme reader. Before lipid staining, the cell count in different groups was assessed based on absorbance at 490 nm by the cell proliferation assay using the Cell Counting Kit-8 (DOJINDO, Kumamoto, Japan), and the cell count was used to normalize the extraction data. The lipid droplet accumulation was presented as a ratio of OD 510/OD 490.

### 2.8. Statistical Analyses

Allele frequencies and genotype frequencies were directly calculated by counting in Microsoft Excel spreadsheets, and differences in allele and genotype frequencies between the fat and lean lines were analyzed using SPSS Statistics software version 26.0. Associations between SNPs and abdominal fat weight, as well as abdominal fat percentage, were analyzed using JMP Statistical Software version 14.0 according to the following model:

Y_ijkl_ = μ + G_i_ + F_j_ + D_k (j)_ + L_l_ + BW_7_ + *e*_ijkl_

Y_ijkl_ represents the phenotypic observation for traits (AFW or AFP), μ is the overall population mean of the traits, G_i_ is the fixed effect of the (i)th genotype, F_j_ is the random effect of the (j)th family, D_k (j)_ is the random effect of the (k)th dam nested within the (j)th family, L_l_ is the fixed effect of the (l)th line, BW_7_ is the covariance variable, and *e*_ijkl_ is the residual random error. In addition, multiple comparisons of least-squares means among individuals with different genotypes were performed for SNPs significantly associated with abdominal fat traits. The differences between the groups were considered significant at *p* < 0.05 (*) and highly significant at *p* < 0.01 (**).

## 3. Results

### 3.1. Identification and Genotyping of SNPs Within the CTGF Gene and Its 3 Kb Flanking Regions

**Figure 1:**
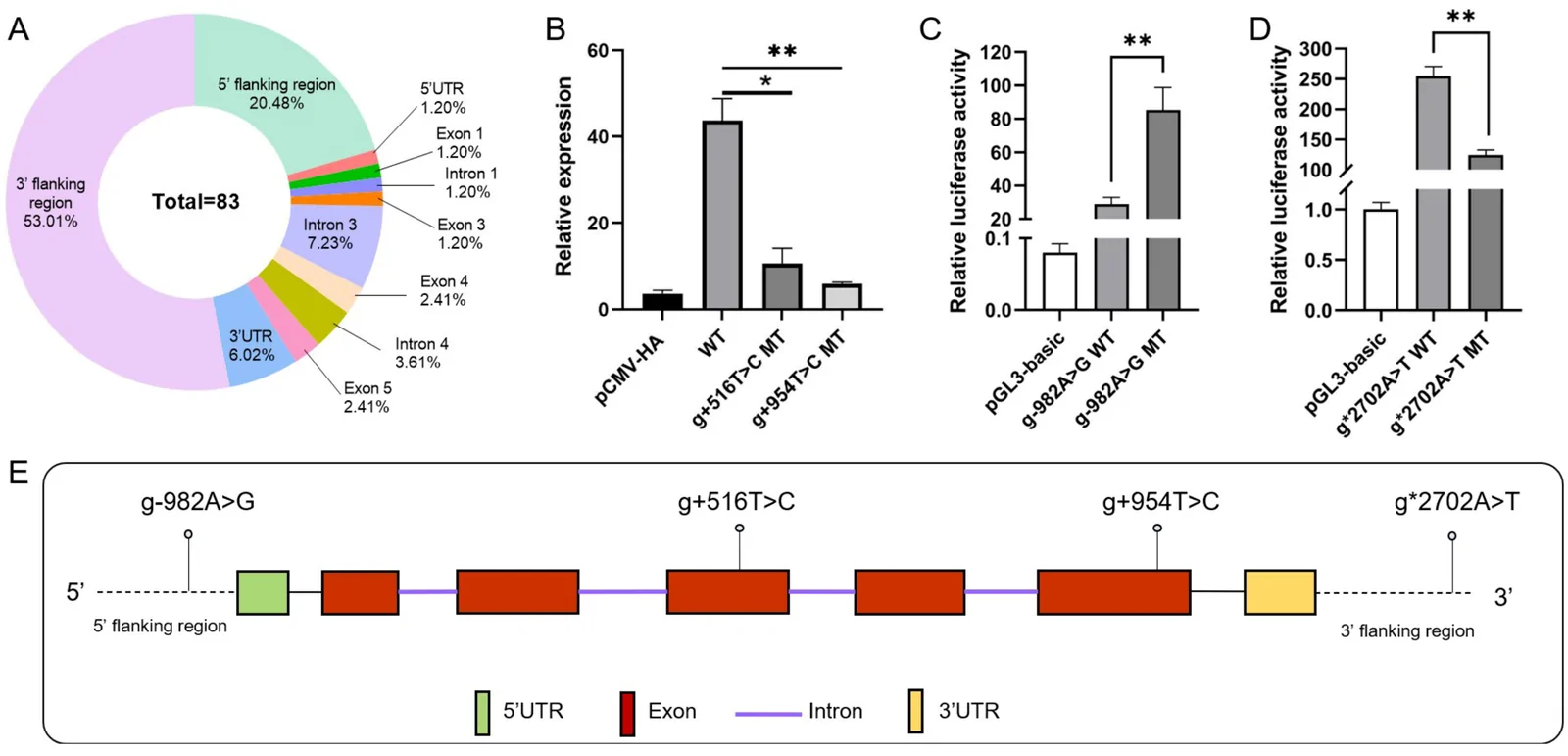
Distribution of SNPs within the entire CTGF gene region and identification of functional SNPs. (**A**) Distribution of SNPs within the 3000 bp upstream and downstream regions of the CTGF gene. (**B**) The mRNA expression level of the CTGF gene. (**C**) Luciferase reporter activities of the WT and MT constructs at the g − 982A > G locus. (**D**) Luciferase reporter activities of the WT and MT constructs at the g *2702A > T locus. (**E**) Distribution of functional SNP loci. Abbreviations: CTGF, connective tissue growth factor; WT, wild-type; MT, mutant-type. ** *p* < 0.01; * *p* < 0.05.

In our previous study, we obtained the whole-genome resequencing data of the 19th generation of fat and lean broiler lines, including 160 lean male broilers and 170 fat male broilers. Based on this data, all SNPs in the CTGF gene and its 3 kb flanking regions were obtained. The results showed that a total of 83 SNPs were identified, including 17 SNPs located in the 5′ flanking region, 44 SNPs in the 3′ flanking region, and 22 SNPs within the CTGF gene. Among the 22 SNPs located within the CTGF gene, 1 SNP was located in the 5′ untranslated region (5′UTR), 6 SNPs were located in exons, 10 SNPs were located in introns, and 5 SNPs were located in the 3′ untranslated region (3′UTR) (Figure 1A). Differences in allele and genotype frequencies between the fat and lean lines were analyzed using a Chi-square test. The results showed that 65 SNPs exhibited significant (*p* < 0.05) or highly significant (*p* < 0.01) differences in allele or genotype frequencies between the two male broiler lines (Table S2).

### 3.2. Association Analysis Between SNPs of CTGF Gene and Abdominal Fat Traits

Association analysis was performed between abdominal fat traits and the 65 selected SNPs that showed significant differences in allele or genotype frequencies between the fat and lean male broiler lines. The results indicated that the polymorphisms of seven SNPs were significantly associated with AFW or AFP in the 19th generation of fat and lean male broilers. These seven SNPs were g − 982A > G, g + 516T > C, g + 954T > C, g *1380A > G, g *2702A > T, g *3319G > A and g *3337T > G (Table 1). Among these SNPs, g − 982A > G was located in the 5′ flanking region, g + 516T > C and g + 954T > C were located in exonic regions, and g *1380A > G, g *2702A > T, g *3319G > A, and g *3337T > G were located in the 3′ flanking region.

Multiple comparisons of least squares means among different genotypes of every SNP significantly associated with abdominal fat traits were further conducted. The results showed that male broilers with the GG genotype at the g − 982A > G locus had significantly lower AFW and AFP than those with the AG genotype. Male broilers with the CC genotype at the g + 516T > C locus had significantly lower AFW and AFP than those with the TC genotype. Male broilers with the TT genotype at the g + 954T > C locus had significantly lower AFW and AFP than those with the TC and CC genotypes. Male broilers with the GG genotype at the g *1380A > G locus had significantly lower AFW and AFP than those with the AG genotype. Male broilers with the TT genotype at the g *2702A > T locus had significantly lower AFW and AFP than those with the AA and AT genotypes. Male broilers with the GA genotype at the g *3319G > A locus had significantly lower AFP than those with the GG and AA genotypes. Male broilers with the TT genotype at the g *3337T > G locus had significantly lower AFW and AFP than those with the TG and GG genotypes. Detailed results of the multiple comparison analysis are shown in Table 2.

### 3.3. Identification of Functional SNPs Associated with Fat in the Chicken CTGF Gene

Since the SNP loci g + 516T > C and g + 954T > C were located in exon 3 and exon 5 of the CTGF gene, respectively, they may regulate abdominal fat deposition in chickens by affecting CTGF mRNA expression levels. Therefore, WT and MT eukaryotic expression vectors containing the g + 516T > C and g + 954T > C loci were constructed, respectively, in which the target sequence of the WT plasmid was the CTGF coding sequence (CDS). These plasmids were transfected into ICP2 cells, and the effects of the two SNPs on CTGF mRNA expression were detected using the RT-qPCR method. The results showed that, compared with the WT, MT at g + 516T > C and g + 954T > C significantly decreased the mRNA expression level of CTGF (Figure 1B). These results suggested that the exonic SNP loci g + 516T > C and g + 954T > C may regulate abdominal fat deposition in broilers by affecting CTGF mRNA expression levels.

In order to evaluate whether the five SNPs located in the non-coding region of CTGF are functional, luciferase reporter assays were performed. Chicken genomic DNA was used as the template to amplify DNA fragments carrying different alleles of g − 982A > G, g *1380A > G, g *2702A > T, g *3319G > A, and g *3337T > G. For each SNP locus, fragments carrying different alleles were separately inserted into the luciferase reporter vector pGL3-Basic. Since the g *3319G > A and g *3337T > G loci are located close to each other, they shared the same WT luciferase reporter vector. WT and MT reporter vectors containing the g − 982A > G, g *1380A > G, g *2702A > T, g *3319G > A, and g *3337T > G loci were co-transfected into ICP2 cells together with the Renilla luciferase vector, respectively. Luciferase activities were measured using a dual-luciferase reporter assay system. The results showed that the relative luciferase activity was significantly (*p* < 0.01) different between constructs carrying different alleles at g − 982A > G and g *2702A > T (Figure 1C,D), whereas no significant differences were observed for g *1380A > G, g *3319G > A, and g *3337T > G (Figure S1). These results indicate that g − 982A > G and g *2702A > T may be functional SNPs associated with abdominal fat deposition in chicken.

### 3.4. Prediction and Validation of Functional SNP Mechanism

**Figure 2:**
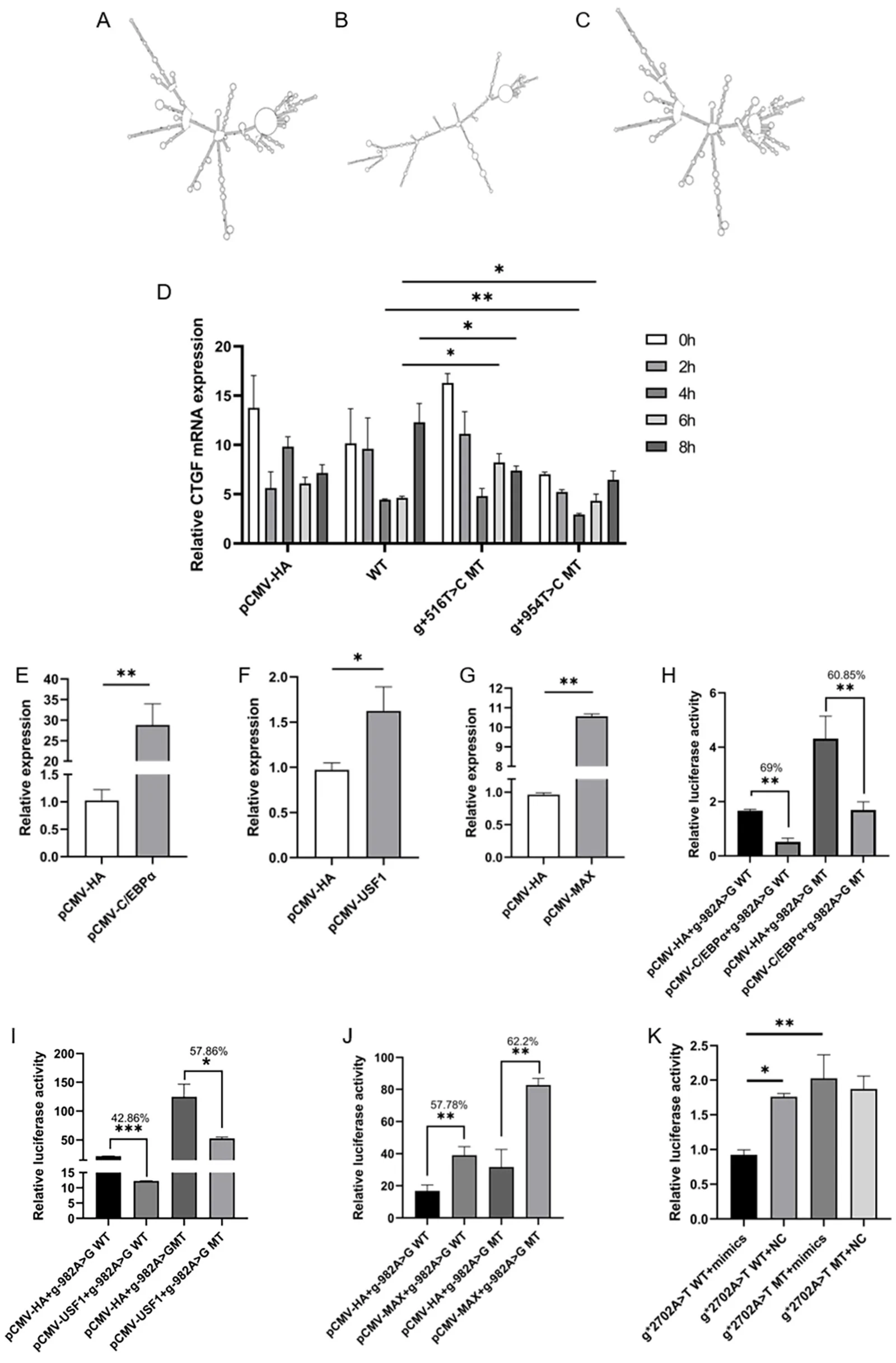
Prediction and validation of functional SNP mechanisms. (**A**–**C**) Prediction of the minimum free energy (MFE) secondary structures of WT and mutant mRNAs at the g + 516T > C and g + 954T > C loci. (**D**) Changes in CTGF mRNA expression levels. (**E**) The mRNA expression level of C/EBPα. (**F**) The mRNA expression level of USF1. (**G**) The mRNA expression level of MAX. (**H**) Effects of the transcription factor C/EBPα on luciferase activities of different alleles at the g − 982A > G locus. (**I**) Effects of the transcription factor USF1 on luciferase activities of different alleles at the g − 982A > G locus. (**J**) Effects of the transcription factor MAX on luciferase activities at the g − 982A > G locus. (**K**) Effects of gga-miR-1812-5p mimics on luciferase reporter activity at the g *2702A > T locus. Abbreviations: CTGF, connective tissue growth factor; C/EBPα, CCAAT/enhancer-binding protein alpha; MAX, MYC-associated factor X; USF1, upstream stimulatory factor 1. *** *p* < 0.001; ** *p* < 0.01; * *p* < 0.05.

First, the potential mechanisms of action of the four functional SNPs identified above were investigated. For g + 516T > C and g + 954T > C, the two exon SNPs were found to be synonymous mutations using the Ensembl gene annotation database. Synonymous mutations may affect gene function by altering mRNA structure, thereby influencing mRNA stability, translation efficiency, or protein expression, as previously reported [17]. Therefore, the effects of g + 516T > C and g + 954T > C mutations on the CTGF mRNA structure were predicted using the RNAfold web server. The results showed that mutations at g + 516T > C and g + 954T > C altered the minimum free energy (MFE) secondary structure of CTGF mRNA (Figure 2A–C). Subsequently, WT and MT vectors containing g + 516T > C and g + 954T > C were transfected into ICP2 cells and treated with 1 mM actinomycin D, respectively. Cells were collected at 0, 2, 4, 6, and 8 h, and CTGF mRNA expression levels were detected by the RT-qPCR method. The results showed that there was a significant (*p* < 0.05) difference in CTGF mRNA expression between WT and MT g + 516T > C at 6 h and 8 h (Figure 2D), indicating that g + 516T > C affects the mRNA stability of the CTGF gene. For g + 954T > C, there was a significant (*p* < 0.05 or *p* < 0.01) difference in the expression of CTGF mRNA between WT and MT at 4 h and 6 h (Figure 2D), indicating that g + 954T > C also affects the mRNA stability of the CTGF gene.

For the SNP g − 982A > G located in the 5′ flanking region, the JASPAR database was used to predict transcription factors capable of binding within the 201 bp region surrounding the SNP locus. The prediction results showed that the sequence carrying the G allele at the g − 982A > G locus contained putative binding sites for CCAAT/enhancer-binding protein alpha (C/EBPα), upstream stimulatory factor 1 (USF1), and MYC-associated factor X (MAX), whereas the sequence carrying the A allele did not. Based on these findings, eukaryotic expression plasmids for C/EBPα, USF1, and MAX were constructed. The Renilla luciferase vector and reporter gene vectors containing A and G alleles of g − 982A > G were co-transfected into ICP2 cells together with the USF1, MAX, or C/EBPα expression vectors, respectively. The luciferase reporter activity results showed that USF1 and C/EBPα significantly (*p* < 0.05 or *p* < 0.01) inhibited reporter gene activity, and this inhibitory effect was regulated by different alleles (Figure 2H,I). After co-transfection with C/EBPα, the activity of the A allele at g − 982A > G decreased by 69%, whereas the activity of the G allele decreased by 60.85% (Figure 2H). After co-transfection with USF1, the activity of the A allele decreased by 42.86%, whereas that of the G allele decreased by 57.86% (Figure 2I). In contrast, the transcription factor MAX promoted reporter gene activity, and this promoting effect was also regulated by different alleles. After co-transfection with MAX, the activity of the A allele increased by 57.78%, whereas that of the G allele increased by 62.2% (Figure 2J).

Previous studies have shown that sequences downstream of gene termini can harbor small-RNA or miRNA recognition sites and participate in gene regulation [18,19]. Therefore, SNPs located in the 3′ flanking region may affect the binding of CTGF to miRNAs. First, the miRDB database was used to predict miRNAs capable of binding to CTGF, and the results identified 65 potential miRNAs targeting CTGF. Subsequently, the RNAhybrid web server was used to predict potential miRNA-binding sites within the 101 bp sequence spanning 50 bp upstream and 50 bp downstream of the g.*2702A > T locus. The analysis identified a potential gga-miR-1812-5p-binding region in the sequence carrying the A allele, beginning at position 46 of the 101 bp target sequence, with a minimum free energy of −17.7 kcal/mol. The g.*2702A > T variant is located at position 51 and therefore lies within the predicted miRNA-binding region. In contrast, no corresponding gga-miR-1812-5p-binding site was predicted for the sequence carrying the T allele. To verify this mechanism, reporter vectors containing different alleles of g *2702A > T were co-transfected into ICP2 cells together with gga-miR-1812-5p mimics. Luciferase reporter assays showed that the WT and MT constructs of g *2702A > T exhibited different responses to gga-miR-1812-5p mimics, resulting in significant differences in reporter activity (*p* < 0.01) (Figure 2K). The above results indicate that four functional SNPs associated with abdominal fat deposition were identified in the chicken CTGF gene, including g − 982A > G, g + 516T > C, g + 954T > C, and g *2702A > T (Figure 1E).

### 3.5. Effects of the g + 516T > C and g + 954T > C Mutations on Chicken Preadipocyte Differentiation

**Figure 3:**
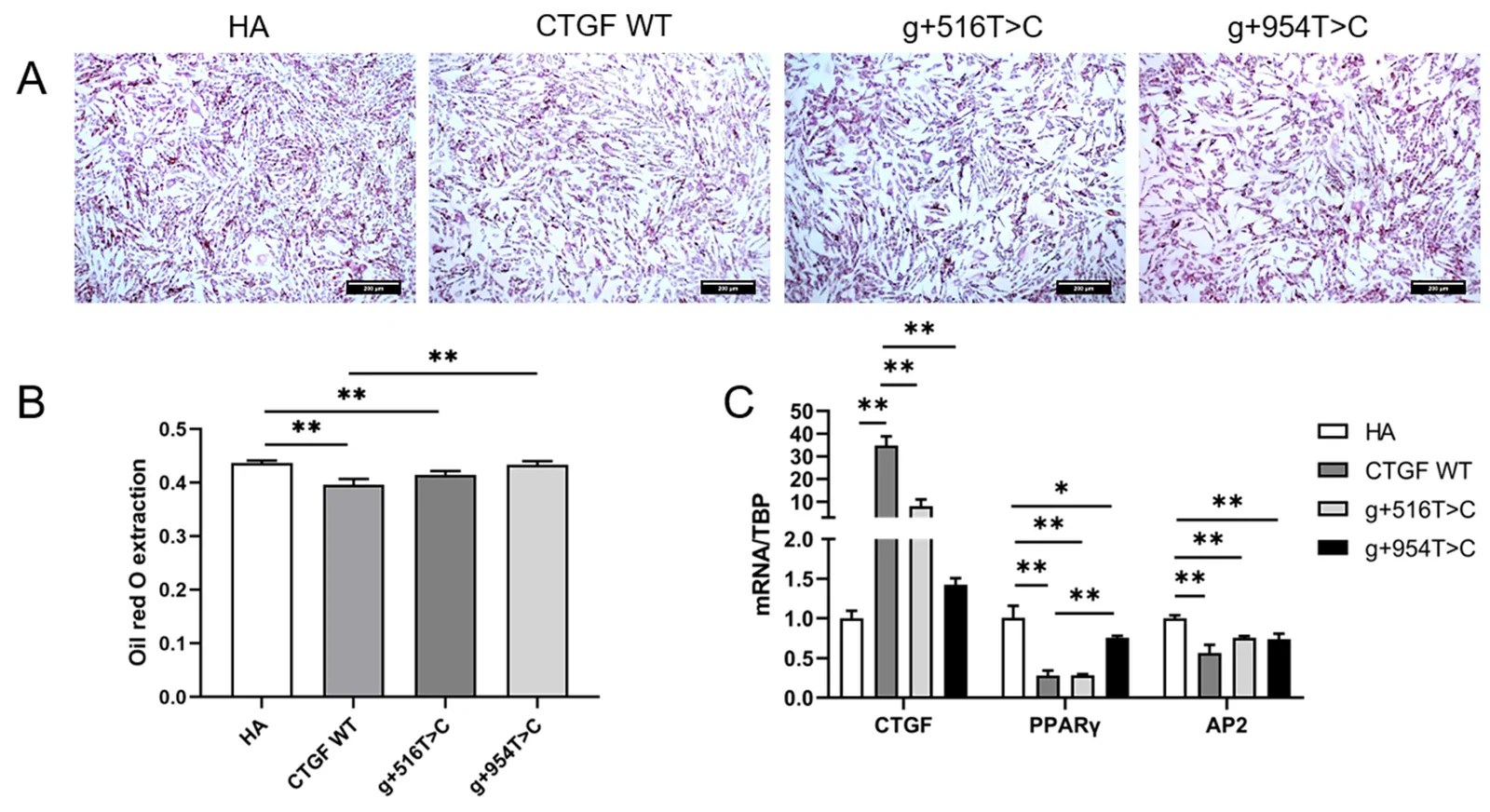
Effects of the g + 516T > C and g + 954T > C mutations on chicken preadipocyte differentiation. (**A**) Oil red oxygen staining. Scale bar: 200 μm. (**B**) Oil red oxygen extraction assay. (**C**) mRNA expression of chicken preadipocyte differentiation marker genes. ** *p* < 0.01; * *p* < 0.05.

In our previous study, we demonstrated that the CTGF gene inhibits the differentiation of chicken preadipocytes [14]. To further investigate the effects of the exonic SNPs g + 516T > C and g + 954T > C on the regulatory function of CTGF during chicken preadipocyte differentiation, eukaryotic expression vectors carrying the WT or MT alleles were transfected into ICP2 cells. Oil red O staining showed that lipid droplet accumulation was increased in cells carrying the g + 954T > C MT compared with the WT group (Figure 3A,B). Consistently, RT-qPCR analysis revealed that the expression level of the marker gene PPARγ was significantly higher in the g + 954T > C MT group than in the WT group (Figure 3C). These results indicated that the g + 954T > C mutation attenuates the inhibitory effect of CTGF on chicken preadipocyte differentiation. In contrast, the g + 516T > C mutation still exhibited an inhibitory effect on chicken preadipocyte differentiation (Figure 3).

## 4. Discussion

The broiler industry plays a critical role in the global supply of animal protein. With the advancement of intensive poultry production systems, feed costs account for nearly 70% of total production expenses, and excessive abdominal fat deposition has become a major economic concern because of its negative impact on feed conversion efficiency [20]. Reducing excessive abdominal fat deposition is beneficial for improving feed efficiency, carcass yield, and the economic value of broiler production. Genetic selection based on functional variants provides an effective strategy for reducing abdominal fat deposition. As important molecular markers in marker-assisted selection, single-nucleotide polymorphisms (SNPs) have been widely investigated in livestock and poultry breeding programs [21,22,23]. In the present study, genetic variants in the CTGF locus are associated with abdominal fat traits in divergently selected broiler lines, suggesting their potential value as molecular markers for the genetic improvement of abdominal fat deposition in broilers.

CTGF is involved in multiple biological processes, including angiogenesis, chondrogenesis, osteogenesis, wound healing, fibrosis, and tumorigenesis [24]. Increasing evidence also indicates that CTGF plays an important role in adipose tissue regulation and adipogenesis. For example, CTGF has been reported to inhibit adipocyte differentiation in mouse preadipocytes, showing altered expression during adipogenesis and obesity-related adipose tissue remodeling [25,26,27]. In this study, seven SNPs associated with abdominal fat traits were found in the CTGF gene and its upstream and downstream 3000 bp. Given that the SNPs examined in this study were located within the same candidate gene region and some were in linkage disequilibrium, the association analysis was primarily intended for exploratory screening and prioritization of candidate variants rather than for establishing independent association signals under stringent multiple-testing correction. Therefore, these associations should be interpreted cautiously and require further validation in larger and independent populations. In addition, AFP was calculated as AFW/BW7, whereas BW7 was also included as a covariate in the association model to account for differences in body size among individuals. Although this adjustment was intended to reduce the potential confounding effect of body weight on abdominal fat traits, the additional adjustment for BW7 may introduce a degree of over-adjustment because BW7 is already incorporated into the calculation of AFP. Consequently, the estimated associations between SNPs and AFP may be partially influenced by the additional adjustment for BW7. Four of them were further confirmed as functional SNPs by reporter gene activity and mRNA stability analysis, which were g + 516T > C, g + 954T > C, g − 982A > G, and g *2702A > T.

Exonic SNPs can be classified into synonymous and nonsynonymous mutations. The major difference between these two MTs is whether the nucleotide substitution results in an amino acid change, which may subsequently alter the physicochemical properties of the encoded protein, such as hydrophobicity and stability, thereby affecting gene function. Although synonymous mutations do not alter the encoded amino acid sequence, increasing evidence suggests that they are not necessarily “silent mutations”, as they may influence gene function by affecting mRNA secondary structure, translational efficiency, codon usage bias, and mRNA stability [28,29,30]. Ensembl database annotation confirmed that the g + 516T > C and g + 954T > C variants within the CTGF gene are authentic synonymous mutations. Therefore, we investigated whether these two SNPs could influence the secondary structure of CTGF mRNA. RNAfold prediction revealed that both g + 516T > C and g + 954T > C substitutions altered the MF secondary structure of CTGF mRNA. Changes in MFE and RNA secondary structure are often associated with altered mRNA stability [31], suggesting that these synonymous mutations may influence CTGF expression at the post-transcriptional level. To verify this possibility, an actinomycin D (ActD) assay was performed in cells transfected with WT or MT CTGF expression plasmids. The results showed significant differences in CTGF mRNA abundance between the WT and MT alleles following transcriptional inhibition. These findings indicate that both synonymous SNPs can influence CTGF mRNA stability. CTGF has previously been shown to inhibit chicken preadipocyte differentiation. In order to further explore the biological significance of these SNPs, WT and MT expression plasmids were transfected into chicken preadipocytes and it was found that these SNPs had a certain inhibitory effect on the differentiation of chicken preadipocytes. In summary, our results indicated that the synonymous mutations g + 516T > C and g + 954T > C were functional variants that alter the secondary structure and stability of CTGF mRNA. These changes may subsequently affect the expression of preadipocyte differentiation marker genes, thereby affecting the differentiation of chicken preadipocytes and the regulation of fat deposition.

SNPs located within promoter or 5′ flanking regions may alter transcription factor-binding affinity, thereby affecting transcriptional activity [32,33]. In this study, transcription factor-binding prediction identified potential C/EBPα-, USF1-, and MAX-binding sites in the sequence carrying the G allele of g.-982A > G. Furthermore, co-transfection assays showed that overexpression of these transcription factors altered reporter activity in an allele-dependent manner. C/EBPα and USF1 inhibited reporter activity, whereas MAX enhanced reporter activity, with different magnitudes of response between the A and G allele constructs. These findings support the possibility that g.-982A > G affects the transcriptional regulatory properties of the CTGF 5′ flanking region. Previous studies have demonstrated that C/EBPα is a key regulator of adipocyte differentiation [23,34]. Many adipocyte-related genes contain C/EBP-responsive elements within their promoter regions and can be transcriptionally activated by C/EBPα. Overexpression of C/EBPα has also been shown to induce adipogenesis in 3T3-L1 preadipocytes [35]. In addition, USF1 deficiency has been reported to activate brown adipose tissue and improve lipid metabolism in mice [36], suggesting that USF1 is involved in adipogenesis and energy metabolism. By contrast, MAX is primarily recognized as a MYC-interacting transcription factor that forms heterodimers with MYC to regulate gene transcription, cell proliferation, and metabolism [37,38], and its role in adipogenesis remains largely unclear. In this study, co-transfection experiments showed that C/EBPα and USF1 significantly inhibited the reporter gene activity at the g − 982A > G locus in an allele-dependent manner, while MAX significantly enhanced the reporter gene activity. These findings suggest that they may be involved in the transcriptional regulation of CTGF. Together, the prediction and co-transfection results suggest that g.-982A > G may influence the transcriptional regulatory response of the CTGF 5′ flanking region to C/EBPα, USF1, and MAX. However, because reporter assays primarily measure transcriptional output rather than direct DNA–protein interactions, these findings should not be interpreted as direct evidence that the variant alters transcription factor-binding affinity. Additional experiments, including EMSA or ChIP-based assays, will be needed to clarify whether the two alleles differ in their ability to recruit these transcription factors. Notably, the direction of the allele-specific reporter activity was consistent with the association results when considering the inhibitory role of CTGF in adipogenesis. The G allele construct showed higher reporter activity than the A allele construct, suggesting a potentially stronger transcriptional regulatory effect on CTGF. Because CTGF acts as a negative regulator of adipogenesis [14], increased CTGF activity would be expected to suppress fat deposition. Conversely, the lower reporter activity associated with the A allele may result in weaker CTGF-mediated inhibition of adipogenesis, which is consistent with the observed association of the A allele with higher AFW and AFP.

MicroRNAs (miRNAs) are small non-coding RNAs approximately 19–22 nucleotides in length that typically bind to the 3′ untranslated region (3′UTR) of target genes, leading to translational repression or mRNA degradation. However, miRNA-mediated regulation is not strictly limited to canonical 3′ UTR regions. Previous studies have shown that sequences downstream of the 3′ termini of protein-coding genes can be enriched with miRNA target sites, and miRNA mimics or small RNAs targeting these regions may participate in gene expression regulation by recognizing non-coding transcripts overlapping gene termini [39]. Therefore, SNPs located in the 3′ flanking region of genes may also affect gene regulation by altering potential miRNA recognition sites [19]. In this study, bioinformatic prediction using miRDB and RNAhybrid indicated that the WT allele of the g *2702A > T locus may bind gga-miR-1812-5p, whereas the mutant allele potentially disrupts this interaction. Numerous studies have demonstrated that miRNAs are closely associated with adipocyte differentiation, lipolysis, glucose uptake, and insulin sensitivity [40,41]. For example, FOXO1 has been shown to inhibit porcine preadipocyte differentiation by suppressing PPARγ expression, and FOXO1 was further identified as a target gene of miR-15a/b [42]. In addition, KLF5, an important regulator of adipogenesis, can be targeted by miR-448 within its 3′UTR, thereby affecting adipocyte differentiation [43]. miR-128-3p inhibits chicken intramuscular adipocyte differentiation by targeting FDPS, miR-23b promotes porcine preadipocyte differentiation by targeting SESN3, and miR-107 suppresses bovine adipocyte differentiation and lipogenesis by targeting APOC2 [44,45,46]. Although no studies have directly reported that miRNA-mediated regulation of CTGF affects adipogenesis, previous studies demonstrated that miR-218 directly targets CTGF and suppresses angiogenesis in colorectal cancer, indicating the existence of a regulatory interaction between miRNAs and CTGF. In the present study, dual-luciferase reporter assays showed that the WT allele of g *2702A > T significantly reduced reporter activity following miRNA co-transfection, whereas the MT allele exhibited significantly increased reporter activity. These findings suggest that the g.*2702A > T locus may affect CTGF regulation by altering a potential miRNA-responsive element. However, the endogenous involvement of gga-miR-1812-5p in CTGF regulation in chicken adipose tissue remains unclear and requires further validation.

## 5. Conclusions

Collectively, four functional SNPs associated with abdominal fat deposition were identified in the chicken CTGF gene, including g − 982A > G, g + 516T > C, g + 954T > C, and g *2702A > T. Interestingly, these functional variants appear to regulate CTGF expression at multiple levels, including transcriptional activation, mRNA stability, and potential miRNA-mediated post-transcriptional regulation. Among them, the g.-982A > G locus may represent a potential regulatory variant affecting CTGF transcriptional activity through allele-dependent responses to transcription factors C/EBPα, whereas the synonymous mutations g + 516T > C and g + 954T > C may affect CTGF function through alterations in mRNA secondary structure and stability. In addition, g.*2702A > T may influence CTGF regulation through a potential miRNA-responsive mechanism. Overall, these findings provide novel insights into the molecular mechanisms underlying fat deposition in broilers and provide potential molecular markers for the genetic improvement of abdominal fat traits.

## Figures and Tables

**Table 1:** Effects (*p*-value) of CTGF polymorphisms on abdominal fat traits (AFW and AFP).

SNP	Chromosomal Position	*p*-Value	
		AFP	AFW
g − 982A > G	Ch3 56835687	0.0676	0.0479
g + 516T > C	Ch3 56837370	0.1229	0.0163
g + 954T > C	Ch3 56838321	0.0031	0.0104
g *1380A > G	Ch3 56839782	0.0398	0.0485
g *2702A > T	Ch3 56841104	0.0303	0.0334
g *3319G > A	Ch3 56841721	0.0240	0.1454
g *3337T > G	Ch3 56841739	0.0056	0.0041

Abbreviations: CTGF, connective tissue growth factor; AFP, abdominal fat percentage; AFW, abdominal fat weight.

**Table 2:** Effects (LSM ± SE) of SNPs on abdominal fat traits.

SNPs	Gene Type (Number of Individuals)	Traits (Unit)	
		AFP (%)	AFW (g)
g − 982A > G	AA (124)	2.897 ± 0.279 ^AB^	56.128 ± 5.311 ^AB^
	AG (80)	2.998 ± 0.280 ^A^	58.234 ± 5.323 ^A^
	GG (126)	2.820 ± 0.279 ^B^	54.378 ± 5.306 ^B^
g + 516T > C	TT (148)	2.878 ± 0.277 ^AB^	55.471 ± 5.270 ^B^
	TC (77)	3.000 ± 0.278 ^A^	59.017 ± 5.293 ^A^
	CC (105)	2.857 ± 0.276 ^B^	54.969 ± 5.240 ^B^
g + 954T > C	TT (157)	2.706 ± 0.283 ^B^	52.601 ± 5.384 ^B^
	TC (98)	2.922 ± 0.281 ^A^	56.453 ± 5.330 ^A^
	CC (75)	2.970 ± 0.279 ^A^	57.445 ± 5.297 ^A^
g *1380A > G	AA (286)	2.893 ± 0.274 ^AB^	55.930 ± 5.192 ^AB^
	AG (31)	3.061 ± 0.289 ^A^	59.604 ± 5.519 ^A^
	GG (13)	2.601 ± 0.315 ^B^	50.693 ± 6.092 ^B^
g *2702A > T	AA (132)	2.961 ± 0.280 ^A^	56.894 ± 5.321 ^A^
	AT (92)	2.952 ± 0.281 ^A^	57.707 ± 5.346 ^A^
	TT (106)	2.778 ± 0.281 ^B^	53.799 ± 5.345 ^B^
g *3319G > A	GG (267)	3.048 ± 0.304 ^A^	57.626 ± 5.856 ^A^
	GA (46)	2.715 ± 0.288 ^B^	53.142 ± 5.503 ^A^
	AA (17)	2.920 ± 0.277 ^A^	56.490 ± 5.255 ^A^
g *3337T > G	TT (137)	2.738 ± 0.281 ^B^	52.660 ± 5.357 ^B^
	TG (103)	2.973 ± 0.280 ^A^	57.382 ± 5.327 ^A^
	GG (90)	2.945 ± 0.279 ^A^	57.335 ± 5.308 ^A^

Note: Different letters indicate significant differences among the three genotypes. Abbreviations: AFP, abdominal fat percentage; AFW, abdominal fat weight; LSM ± SE, least squares means ± standard error.

## Data Availability

None of the data were deposited in an official repository. Data are available from the authors upon request.

## Supplementary Materials

The following supporting information can be downloaded at https://www.mdpi.com/article/10.3390/cells15211930/s1: Figure S1: Identification of functional SNPs of CTGF; Table S1: Sequences of primers used in this study; Table S2: Allele frequency distribution of SNPs in the CTGF gene and its flanking regions.
