# Supplementary material for: Identification of Functional SNPs in CTGF Regulating Fat Deposition in Broilers
Source: Cells. 2026 Oct 23;15(21):1930. doi: 10.3390/cells15211930 (PMC13761654; doi:10.3390/cells15211930)
Supplement: Supplementary file 1 [file cells-15-01930-s001.zip › cells-4607876-supplementary/Supplementary materials.pdf]

Table S1. Sequences of primers used in this study

Primer	Primer sequence (5′→3′)
*CTGF*5687W	F: CTCGAACacaAGCAGACCGCAGCGTTTGGGTT
	R: GTCTGCTtgtGTTCGAGCCCCAAACCTGTGGT
*CTGF*9782W	F: gcgtgctagcccgggctcgagTGGCCTGATATCTCGTTTATCTTCA
	R: cagtaccggaatgccaagcttCCCATGAAGCTTAAATCAGAAGATC
*CTGF*1104W	F: gctagcccgggctcgagatctAGAGGAACAAAAAAAAACACAGGAG
	R: cagtaccggaatgccaagcttCTCTTCCTACTTTTCTTCTGGCCT
*CTGF*1721W	F: CATCTCACAgGCATTAGACTTCTGAAGTTTTTTACCTC
	R: CTAATGCcTGTGAGATGTGTAAAATGCGTGCT
*CTGF*1739W	F: CTCActttTTTACCTCTGAAGGTAAAGAAATTTAAATC
	R: CAGAGGTAAAaaagTGAGATGTGTAAAATGCGTGCTT
*CTGF*1739M	F: CTGAAGgTTTTTACCTCTGAAGGTAAAGAAATTTAA
	R: GAGGTAAAAAcCTTCAGAAGTCTAATGCCTGGAGA
*CTGF*56M	F: TCGAACACgAGCAGACCGCAGCGTTTGGGTTT
	R: GGTCTGCTCGTGTTCGAGCCCCAAACCTGTGG
*CTGF*7370MF	F: tgggacccgcactggccgCCTACAGACTGGAAGACACTTACGG
	R: cggccagtgcgggtcccaCCGCCGTCTGCTCCTTGG
*CTGF*8321M	F: TTCATCAAGACCTGcGCCTGCCACTACAACTGCCC
	R: GCgCAGGTCTTGATGAACATCATTTTCCTTTT
*CTGF*11M	F: ACCTACACtGTGTTTTTTTTAAAGTGGTCAAAACTG
	R: AAAAACACaGTGTAGGTTTTTAATCAATAGGTTTGAAC
*CTGF*97M	F: ACCCACTgTAACAATCCATGCAATGTGCTTAG
	R: GATTGTTAcAGTGGGGTTGAATACTGCAGTGA
pCMV-*USF1*	F: gccatggaggcccgaattcGGATGAAGGGACAGCAGAAAGCAG
	R: tccccgcggccgcggtaccTCAGTGGCTCTCGTTCTTGATGAC
pCMV-*MAX*	F: tggccatggaggcccgaattcGGATGAGCGACAACGATGACA
	R: ccgcggtacctcgagagatctCTAGCTGGCCTCCATGCGC
C/EBPα	F: GCGACATCTGCGAGAACG
	R: GTACAGCGGGTCGAGCTT
USF1	F: TGTCGTCACCACGCAGAACTCA
	R: TGTGCCCGACGCTTCTCATC
MAX	F: AAACGAGCCCACCACAACGC
	R: GGCGCATGTACTGGATGTACTCTG
CTGF	F: ATCTCGACCAGGGTCACCAAC
	R: GTGCAGCCAGACAGCTCAAAC

PPARγ	F: GTGCAATCAAAATGGAGCC
	R: CTTACAACCTTCACATGCAT
AP2	F: ATGTGCGACCAGTTTGT
	R: TCACCATTGATGCTGATAG
TBP	F: GCGTTTTGCTGCTGTTATTATGAG
	R: TCCTTGCTGCCAGTCTGGAC

Table S2. Allele Frequency Distribution of SNPs in the CTGF Gene and Its Flanking Regions

SNPs	Lines	Genotype frequencies (No. of birds)			χ^2	Allele frequencies		χ^2
		AA	AG	GG		A	G	
g-2265A>G	fat	0.038 (6)	0.000 (0)	0.962 (154)	20.200	0.038	0.962	9.931
	lean	0.041 (7)	0.118 (20)	0.841 (143)	(*P*<0.01)	0.100	0.900	(*P*<0.01)
		AA	AG	GG		A	G	
g-2240A>G	fat	0.038 (6)	0.000 (0)	0.962 (154)	20.382	0.038	0.962	16.241
	lean	0.071 (12)	0.106 (18)	0.823 (140)	(*P*<0.01)	0.124	0.876	(*P*<0.01)
		GG	GA	AA		G	A	
g-2143G>A	fat	0.056 (9)	0.000 (0)	0.944 (151)	21.000	0.056	0.944	13.954
	lean	0.088 (15)	0.112 (19)	0.80 (136)	(*P*<0.01)	0.144	0.856	(*P*<0.01)
		GG	GA	AA		G	A	
g-1254G>A	fat	0.819 (131)	0.156 (25)	0.025 (4)	15.749	0.897	0.103	17.926
	lean	0.953 (162)	0.047 (8)	0.000 (0)	(*P*<0.01)	0.976	0.024	(*P*<0.01)
		AA	AG	GG		A	G	
g-1163A>G	fat	0.800 (128)	0.175 (28)	0.025 (4)	24.422	0.887	0.113	27.060
	lean	0.971 (165)	0.029 (5)	0.000 (0)	(*P*<0.01)	0.985	0.015	(*P*<0.01)
		CC	CG	GG		C	G	
g-1159A>G	fat	0.794 (127)	0.171 (29)	0.025 (4)	25.067	0.880	0.120	28.176
	lean	0.971 (165)	0.029 (5)	0.000 (0)	(*P*<0.01)	0.985	0.015	(*P*<0.01)
		CC	CT	TT		C	T	
g-1134C>T	fat	0.794 (127)	0.169 (27)	0.037 (6)	25.791	0.879	0.121	30.428

	lean	0.971 (165)	0.029 (5)	0.000 (0)	($P<0.01$)	0.985	0.015	($P<0.01$)
		GG	GT	TT		G	T	
g-1033G>T	fat	0.306 (49)	0.35 (56)	0.344 (55)	22.014	0.481	0.519	29.151
	lean	0.547 (93)	0.282 (48)	0.171 (29)	($P<0.01$)	0.688	0.312	($P<0.01$)
		AA	AG	GG		A	G	
g-982A>G	fat	0.213 (34)	0.256 (41)	0.531 (85)	50.883	0.341	0.659	73.121
	lean	0.529 (90)	0.288 (49)	0.182 (31)	($P<0.01$)	0.673	0.327	($P<0.01$)
		CC	CT	TT		C	T	
g-942C>T	fat	0.844 (135)	0.106 (17)	0.050 (8)	14.513	0.897	0.103	19.725
	lean	0.965 (164)	0.029 (5)	0.006 (1)	($P<0.01$)	0.979	0.021	($P<0.01$)
		AA	AC	CC		A	C	
g-890A>C	fat	0.444 (71)	0.225 (36)	0.331 (53)	9.698	0.556	0.444	9.592
	lean	0.529 (90)	0.288 (49)	0.183 (31)	($P<0.01$)	0.673	0.327	($P<0.01$)
		CC	CT	TT		C	T	
g-614C>T	fat	0.794 (127)	0.113 (18)	0.093 (15)	25.825	0.851	0.149	38.439
	lean	0.971 (165)	0.024 (4)	0.005 (1)	($P<0.01$)	0.983	0.017	($P<0.01$)
		CC	CT	TT		C	T	
g-562C>T	fat	1.000 (160)	0.000 (0)	0.000 (0)	6.731	1.000	0.000	7.622
	lean	0.959 (163)	0.035 (6)	0.006 (1)	($P=0.035$)	0.976	0.024	($P<0.01$)
		CC	CT	TT		C	T	
g-304C>T	fat	0.844 (135)	0.063 (10)	0.093 (15)	6.853	0.875	0.125	5.842
	lean	0.735 (125)	0.141 (24)	0.124 (21)	($P=0.033$)	0.805	0.195	($P=0.016$)
		AA	AG	GG		A	G	

g-181A>G	fat	0.588 (94)	0.225 (36)	0.187 (30)	54.869	0.700	0.300	79.511
	lean	0.924 (157)	0.071 (12)	0.005 (1)	(*P*<0.01)	0.959	0.041	(*P*<0.01)
		CC	CA	AA		C	A	
g-32C>A	fat	1.000 (160)	0.000 (0)	0.000 (0)	9.706	1.000	0.000	13.462
	lean	0.941 (160)	0.035 (6)	0.024 (4)	(*P*<0.01)	0.959	0.041	(*P*<0.01)
		CC	CT	TT		C	T	
g+21C>T	fat	0.681 (109)	0.225 (36)	0.094 (15)	64.093	0.793	0.207	77.917
	lean	1.000 (170)	0.000 (0)	0.000 (0)	(*P*<0.01)	1.000	0.000	(*P*<0.01)
		TT	TC	CC		T	C	
g52+5T>C	fat	0.406 (65)	0.244 (39)	0.35 (56)	26.780	0.528	0.472	29.608
	lean	0.576 (98)	0.312 (53)	0.112 (19)	(*P*<0.01)	0.732	0.268	(*P*<0.01)
		TT	TC	CC		T	C	
g+516T>C	fat	0.319 (51)	0.213 (34)	0.468 (75)	34.363	0.425	0.575	60.577
	lean	0.571 (97)	0.253 (43)	0.176 (30)	(*P*<0.01)	0.698	0.302	(*P*<0.01)
		GG	GA	AA		G	A	
g526+35G>A	fat	1.000 (160)	0.000 (0)	0.000 (0)	13.760	1.000	0.000	16.423
	lean	0.918 (156)	0.065 (11)	0.017 (3)	(*P*<0.01)	0.950	0.050	(*P*<0.01)
		GG	GA	AA		G	A	
g526+84G>A	fat	0.896 (139)	0.100 (16)	0.031 (5)	10.135	0.919	0.081	12.771
	lean	0.959 (163)	0.041 (7)	0.000 (0)	(*P*<0.01)	0.980	0.020	(*P*<0.01)
		CC	CT	TT		C	T	
g527-86C>T	fat	0.681 (109)	0.244 (39)	0.075 (12)	64.093	0.803	0.197	74.001

	lean	1.000 (170)	0.000 (0)	0.000 (0)	($P<0.01$)	1.000	0.000	($P<0.01$)
		GG	GA	AA		G	A	
g527-11G>A	fat	0.644 (103)	0.281 (45)	0.075 (12)	73.207	0.784	0.216	81.872
	lean	1.000 (170)	0.000 (0)	0.000 (0)	($P<0.01$)	1.000	0.000	($P<0.01$)
		CC	CT	TT		C	T	
g+654C>T	fat	1.000 (160)	0.000 (0)	0.000 (0)	13.760	1.000	0.000	15.433
	lean	0.918 (156)	0.071 (12)	0.011 (2)	($P<0.01$)	0.953	0.047	($P<0.01$)
		GG	GA	AA		G	A	
g+661G>A	fat	1.000 (160)	0.000 (0)	0.000 (0)	12.737	1.000	0.000	14.446
	lean	0.924 (157)	0.065 (11)	0.011 (2)	($P<0.01$)	0.956	0.044	($P<0.01$)
		AA	AG	GG		A	G	
g738+91A>G	fat	0.606 (97)	0.288 (46)	0.106 (17)	82.732	0.750	0.250	96.724
	lean	1.000 (170)	0.000 (0)	0.000 (0)	($P<0.01$)	1.000	0.000	($P<0.01$)
		GG	GA	AA		G	A	
g738+131G>A	fat	1.000 (160)	0.000 (0)	0.000 (0)	11.270	1.000	0.000	12.481
	lean	0.929 (158)	0.065 (11)	0.006 (1)	($P<0.01$)	0.962	0.038	($P<0.01$)
		TT	TC	CC		T	C	
g738+153T>C	fat	0.125 (20)	0.338(54)	0.537 (86)	59.847	0.294	0.706	78.511
	lean	0.465 (79)	0.347 (59)	0.188 (32)	($P<0.01$)	0.638	0.362	($P<0.01$)
		TT	TC	CC		T	C	
g+954T>C	fat	0.338 (54)	0.337 (54)	0.325 (52)	27.249	0.507	0.493	36.869
	lean	0.606 (103)	0.259 (44)	0.135 (23)	($P<0.01$)	0.736	0.264	($P<0.01$)
		GG	GA	AA		G	A	

g+1024G>A	fat	0.275 (44)	0.381 (61)	0.344 (55)	38.029	0.465	0.535	48.992
	lean	0.588 (100)	0.288 (49)	0.124 (21)	(*P*<0.01)	0.732	0.268	(*P*<0.01)
		AA	AT	TT		A	T	
g*990A>T	fat	0.931 (149)	0.000 (0)	0.069 (11)	12.967	0.931	0.069	21.626
	lean	0.994 (169)	0.006 (1)	0.000 (0)	(*P*<0.01)	0.997	0.003	(*P*<0.01)
		TT	TC	CC		T	C	
g*1078T>C	fat	0.588 (94)	0.119 (19)	0.293 (47)	73.093	0.647	0.353	125.533
	lean	0.971 (165)	0.024 (4)	0.005 (1)	(*P*<0.01)	0.983	0.017	(*P*<0.01)
		TT	TC	CC		T	C	
g*1141T>C	fat	0.594 (95)	0.169 (27)	0.237 (38)	70.775	0.678	0.322	110.661
	lean	0.971 (165)	0.024 (4)	0.006 (1)	(*P*<0.01)	0.983	0.017	(*P*<0.01)
		GG	GT	TT		G	T	
g*1161G>T	fat	0.506 (81)	0.188 (30)	0.306 (49)	30.703	0.600	0.400	49.358
	lean	0.782 (133)	0.124 (21)	0.094 (16)	(*P*<0.01)	0.844	0.156	(*P*<0.01)
		CC	CT	TT		C	T	
g*1296C>T	fat	0.650 (104)	0.263 (42)	0.087 (14)	71.661	0.781	0.219	83.199
	lean	1.000 (170)	0.000 (0)	0.000 (0)	(*P*<0.01)	1.000	0.000	(*P*<0.01)
		CC	CG	GG		C	G	
g*1313C>G	fat	0.481 (77)	0.294 (47)	0.225 (36)	86.920	0.628	0.372	117.388
	lean	0.941 (160)	0.047 (8)	0.012 (2)	(*P*<0.01)	0.964	0.036	(*P*<0.01)
		AA	AG	GG		A	G	
g*1372A>G	fat	0.481 (77)	0.325 (52)	0.194 (31)	78.164	0.643	0.357	94.784

	lean	0.924 (157)	0.047 (8)	0.029 (5)	(*P*<0.01)	0.947	0.053	(*P*<0.01)
		AA	AG	GG		A	G	
g*1380A>G	fat	0.788 (126)	0.144 (23)	0.068 (11)	17.244	0.860	0.140	23.179
	lean	0.941 (160)	0.047 (8)	0.012 (2)	(*P*<0.01)	0.965	0.035	(*P*<0.01)
		AA	AG	GG		A	G	
g*1545A>G	fat	1.000 (160)	0.000 (0)	0.000 (0)	11.270	1.000	0.000	13.462
	lean	0.929 (158)	0.059 (10)	0.012 (2)	(*P*<0.01)	0.959	0.041	(*P*<0.01)
		CC	CG	GG		C	G	
g*2231C>G	fat	0.344 (55)	0.462 (74)	0.194 (31)	61.866	0.575	0.425	69.277
	lean	0.765 (130)	0.2 (34)	0.035 (6)	(*P*<0.01)	0.865	0.135	(*P*<0.01)
		AA	AG	GG		A	G	
g*2237A>G	fat	0.344 (55)	0.456 (73)	0.200 (32)	62.164	0.572	0.428	70.540
	lean	0.765 (130)	0.200 (34)	0.035 (6)	(*P*<0.01)	0.865	0.135	(*P*<0.01)
		GG	GA	AA		G	A	
g*2249G>A	fat	0.344 (55)	0.469 (75)	0.187 (30)	60.789	0.578	0.422	66.406
	lean	0.523 (89)	0.318 (54)	0.159 (27)	(*P*<0.01)	0.682	0.318	(*P*<0.01)
		CC	CT	TT		C	T	
g*2274C>T	fat	0.250 (40)	0.419 (67)	0.331 (53)	29.579	0.459	0.541	35.382
	lean	0.529 (90)	0.318 (54)	0.153 (26)	(*P*<0.01)	0.688	0.312	(*P*<0.01)
		GG	GA	AA		G	A	
g*2462G>A	fat	0.244 (39)	0.275 (44)	0.481 (77)	39.900	0.381	0.619	58.907
	lean	0.559 (95)	0.241 (41)	0.200 (34)	(*P*<0.01)	0.680	0.320	(*P*<0.01)
		TT	TC	CC		T	C	

g*2518T>C	fat	0.588 (94)	0.25 (40)	0.162 (26)	59.662	0.713	0.287	81.482
	lean	0.941 (160)	0.053 (9)	0.006 (1)	(*P*<0.01)	0.968	0.032	(*P*<0.01)
		CC	CG	GG		C	G	
g*2583C>G	fat	0.519 (83)	0.263 (42)	0.218 (35)	22.208	0.650	0.350	27.748
	lean	0.712 (121)	0.235 (40)	0.053 (9)	(*P*<0.01)	0.830	0.170	(*P*<0.01)
		CC	CG	GG		C	G	
g*2637C>G	fat	0.262 (42)	0.219 (35)	0.519 (83)	39.896	0.372	0.628	55.513
	lean	0.518 (88)	0.288 (49)	0.194 (33)	(*P*<0.01)	0.662	0.338	(*P*<0.01)
		TT	TC	CC		T	C	
g*2643C>G	fat	0.262 (42)	0.250 (40)	0.488 (78)	36.445	0.387	0.613	53.079
	lean	0.535 (91)	0.271 (46)	0.194 (33)	(*P*<0.01)	0.670	0.330	(*P*<0.01)
		TT	TC	CC		T	C	
g*2675T>C	fat	0.281 (45)	0.244 (39)	0.475 (76)	28.886	0.403	0.597	42.314
	lean	0.523 (89)	0.265 (45)	0.212 (36)	(*P*<0.01)	0.656	0.344	(*P*<0.01)
		AA	AT	TT		A	T	
g*2702A>T	fat	0.281 (45)	0.225 (36)	0.494 (79)	42.957	0.393	0.607	53.035
	lean	0.512 (87)	0.329 (56)	0.159 (27)	(*P*<0.01)	0.676	0.324	(*P*<0.01)
		AA	AG	GG		A	G	
g*2749A>G	fat	0.225 (36)	0.262 (42)	0.513 (82)	51.469	0.356	0.644	70.287
	lean	0.523 (89)	0.318 (54)	0.159 (27)	(*P*<0.01)	0.682	0.318	(*P*<0.01)
		AA	AG	GG		A	G	
g*2828A>G	fat	0.194 (31)	0.275 (44)	0.531 (85)	59.967	0.331	0.669	84.109

	lean	0.535 (91)	0.306 (52)	0.159 (27)	(*P*<0.01)	0.688	0.312	(*P*<0.01)
		CC	CG	GG		C	G	
g*2830C>G	fat	0.194 (31)	0.275 (44)	0.531 (85)	59.967	0.331	0.669	84.109
	lean	0.535 (91)	0.306 (52)	0.159 (27)	(*P*<0.01)	0.688	0.312	(*P*<0.01)
		CC	CT	TT		C	T	
g*2837C>T	fat	0.200 (32)	0.269 (43)	0.531 (85)	58.062	0.335	0.665	82.659
	lean	0.541 (92)	0.294 (50)	0.165 (28)	(*P*<0.01)	0.688	0.312	(*P*<0.01)
		TT	TC	CC		T	C	
g*2930T>C	fat	0.175 (28)	0.250 (40)	0.575 (92)	66.066	0.300	0.700	90.588
	lean	0.506 (86)	0.329 (56)	0.165 (28)	(*P*<0.01)	0.670	0.330	(*P*<0.01)
		GG	GA	AA		G	A	
g*2939G>A	fat	0.769 (123)	0.169 (27)	0.062 (10)	27.081	0.853	0.147	34.996
	lean	0.959 (163)	0.041 (7)	0.000 (0)	(*P*<0.01)	0.979	0.021	(*P*<0.01)
		CC	CT	TT		C	T	
g*3069C>T	fat	0.588 (94)	0.350 (56)	0.062 (10)	7.767	0.763	0.237	5.944
	lean	0.729 (124)	0.218 (37)	0.053 (9)	(*P*=0.021)	0.838	0.162	(*P*=0.015)
		GG	GC	CC		G	C	
g*3210G>C	fat	0.594 (95)	0.337 (54)	0.069 (11)	86.002	0.763	0.237	91.259
	lean	1.000 (170)	0.000 (0)	0.000 (0)	(*P*<0.01)	1.000	0.000	(*P*<0.01)
		CC	CT	TT		C	T	
g*3268C>T	fat	0.606 (97)	0.319 (51)	0.075 (12)	82.732	0.765	0.235	89.904
	lean	1.000 (170)	0.000 (0)	0.000 (0)	(*P*<0.01)	1.000	0.000	(*P*<0.01)
		GG	GA	AA		G	A	

g*3319G>A	fat	0.606 (97)	0.288 (46)	0.106 (17)	82.732	0.750	0.250	96.724
	lean	1.000 (170)	0.000 (0)	0.000 (0)	(*P*<0.01)	1.000	0.000	(*P*<0.01)
		TT	TG	GG		T	G	
g*3337T>G	fat	0.269 (43)	0.350 (56)	0.381 (61)	30.875	0.444	0.556	41.204
	lean	0.553 (94)	0.276 (47)	0.171 (29)	(*P*<0.01)	0.691	0.309	(*P*<0.01)
		CC	CT	TT		C	T	
g*3573C>T	fat	0.225 (36)	0.244 (39)	0.531 (85)	52.432	0.347	0.653	73.013
	lean	0.535 (91)	0.294 (50)	0.171 (29)	(*P*<0.01)	0.682	0.318	(*P*<0.01)
		CC	CT	TT		C	T	
g*3838C>T	fat	0.331 (53)	0.306 (49)	0.363 (58)	16.281	0.484	0.516	22.712
	lean	0.541 (92)	0.253 (43)	0.206 (35)	(*P*<0.01)	0.668	0.332	(*P*<0.01)
		CC	CT	TT		C	T	
g*3896C>T	fat	0.813 (130)	0.162 (26)	0.025 (4)	3.775	0.894	0.106	3.171
	lean	0.765 (130)	0.165 (28)	0.070 (12)	(*P*=0.151)	0.847	0.153	(*P*=0.075)
		CC	CT	TT		C	T	
g*4034C>T	fat	1.000 (160)	0.000 (0)	0.000 (0)	6.731	1.000	0.000	8.588
	lean	0.959 (163)	0.029 (5)	0.012 (2)	(*P*=0.035)	0.973	0.027	(*P*<0.01)

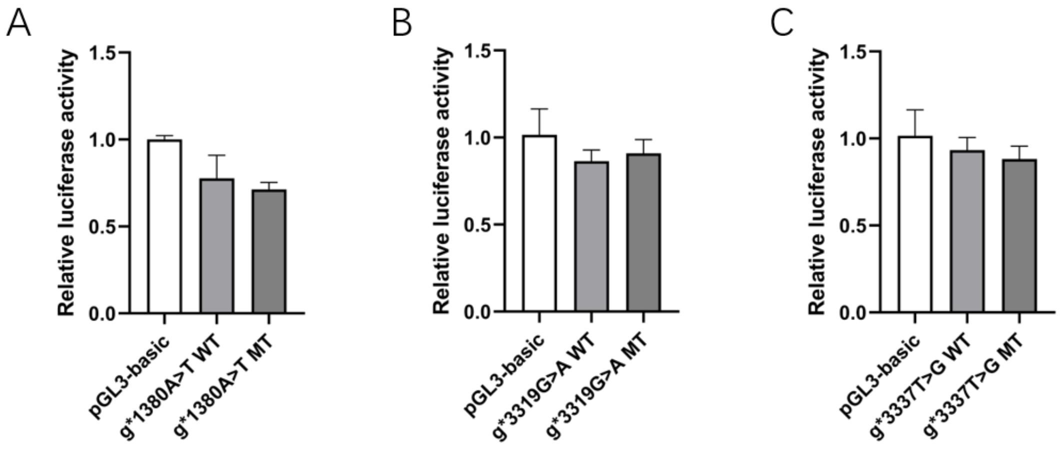

Figure S1. Identification of functional SNPs of *CTGF*. (A) Luciferase reporter activities of the wild-type and mutant constructs at the g*1380A>T locus. (B) Luciferase reporter activities of the wild-type and mutant constructs at the g*3319G>A locus. (C) Luciferase reporter activities of the wild-type and mutant constructs at the g*3337T>G locus.
